# Measured and predicted freeze-thaw days frequencies in climate change conditions in central Poland

**DOI:** 10.7717/peerj.12153

**Published:** 2021-10-26

**Authors:** Arkadiusz Bartczak, Halina Kaczmarek, Michał Badocha, Michał Krzemiński, Sebastian Tyszkowski

**Affiliations:** 1 Department of Environmental Resources and Geohazards, Institute of Geography and Spatial Organization, Polish Academy of Sciences, Toruń, Poland; 2 Department of Landscape Geography, Institute of Geography, Kazimierz Wielki University, Bydgoszcz, Poland; 3 Department of Physical Chemistry, Faculty of Chemistry, Gdańsk University of Technology, Gdańsk, Poland; 4 Institute of Applied Mathematics, Faculty of Applied Physics and Mathematics, Gdańsk University of Technology, Gdańsk, Poland; 5 Department of Environmental Resources and Geohazards, Institute of Geography and Spatial Organization Polish Academy of Sciences, Toruń, Poland

**Keywords:** Freeze-thaw processes, Cliff recession, Prediction of FTD, Central Poland, Polynomial function

## Abstract

The rate of progression of geomorphological phenomena is greatly influenced by freeze-thaw processes. In the face of air temperature increasing over the past few decades, a question of the future impact of these processes arises, notably in the temperate and cold climate zones. Using the mean, maximum and minimum daily air temperature data in the period 1951–2018 obtained from three weather stations located in the vicinity of Jeziorsko reservoir (central Poland), we have determined the mathematical correlation, described with a polynomial function, between the mean monthly air temperature and the monthly number of freeze-thaw days (FTD). A freeze-thaw day is a day when the maximum air temperature is above 0 °C while the minimum air temperature equals or is below this threshold. The number of FTDs within the study area averaged 64–71 and demonstrated a downward trend of 2–4 FTDs/10 years. The study period (1951–2018), includes a clearly marked distinct sub-period (1991–2018), when the reservoir was in operation, which experienced 58–68 FTDs. Considering the assumed rise in temperature, one should expect a further, though slightly slower, decline in the future number of FTDs. Depending on the accepted model of the temperature increase, which for the area of Poland (Central Europe) in the perspective of 30 years oscillates between +1.1 to +1.3 °C, the number of FTDs within the study area is expected to decline by −4.5 to −5.3 FTD, *i.e*. 6–7% and 5.4–5.5 FTD *i.e*. 8–9% respectively.

## Introduction

Owing to its high applicability, research into freeze-thaw processes is valued in various fields of earth and environmental sciences, such as geology, soil science, physics, geomorphology, agrometeorology and agroclimatology or geo- and civil engineering. The temperature of 0 °C is a threshold whose transition triggers processes which considerably alter the properties of individual elements of the environment. These phenomena are brought about by the physical properties of water whose volume expands by 9% as it freezes. Freeze-thaw events are particularly common in mid and high latitudes (Persson et al., 2007; Henry, 2008; Kjellström et al., 2016; Rasmus et al., 2020) and are the most frequent in months with the mean temperature near 0 °C (Baker & Ruschy, 1995; Dewez et al., 2015; Kerguillec, 2015).

Freeze and thaw processes tend to change the physical and mechanical properties of the surface layer of sediments (Jabro et al., 2014) by weakening friction between particles and increasing the content of water in pores while thawing. The potency of the process strongly depends on the type and moisture of the soil, as well as on the speed of freezing and thawing (Mostaghimi et al., 1988). Silts are the most vulnerable to freeze-thaw processes followed by fine sands and clay sediments (Gatto, 1995; Michalowski & Zhu, 2006). Moreover, the influence of freeze-thaw cycles varies depending on the slope of terrain, its exposition and cover (Hall, 2004).

Sediments found on vertical or nearly vertical walls deprived of the protective plant or snow cover are especially vulnerable to freeze-thaw processes. This type of formation is characteristic of *e.g*. coastal systems where they form erosion-prone banks of rivers or reservoirs. Cliffs develop and recede as a consequence of multifactorial processes including freezing and thawing. The emergence and development of coastal cliffs near artificial reservoirs is triggered and propelled mostly by wave erosion as the base of the slopes surrounding a body of water gets undercut (Joeckel & Diffendal, 2004; Brown et al., 2005; Davidson-Arnott, 2016; Kaczmarek et al., 2016). At the same time, the surface of the transformed slopes is subject to subaerial geomorphological processes which strongly promote the development of cliffs. Heavily dependent on local geological and climate conditions, such subaerial processes are frequently modified by wave erosion as to their type and course (Joeckel & Diffendal, 2004; Brown et al., 2005; Kaczmarek, Tyszkowski & Banach, 2015; Kaczmarek et al., 2016; Tyszkowski et al., 2015; Davidson-Arnott, 2016). What is more, the processes often coincide and interact with one another making it impossible to indicate their individual impact on the transformation of the slopes. Exposed to the combined influence of wave erosion and subaerial processes, the material from the cliff face tends to fall off, slide or slump. Then, deposited at the cliff base, the material is systematically moved towards the water by wave action, leading to further destabilisation of the cliff (Carter & Guy, 1988; Spanilá & Simeonova, 1993; Edil, 2010).

As a consequence of the joint “attack” by wave erosion and subaerial geomorphological processes, the cliff undergoes constant morphological transformations resulting in its successive retreat. This process is quantitatively expressed by the magnitude of the linear cliff shift or by the amount of the failed material.

Subjected to repeated freeze-thaw processes, exposed sediments on a cliff face can become incohesive. This process is further facilitated by the appearance of macro cracks in the sediment amplifying the effect of cliff erosion (Kimiaghalam et al., 2015).

Repeated cycles of freeze-thaw and dehydration create a five to 10 mm-thick mechanically weathered surface layer on the face of the cliff which will be removed or will fall off. Consequently, successive layers of sediments become exposed and subjected to freeze-thaw activity leading to the progression of a frost front into cliff sediments and an increase in the cliff retreat (Bernatchez, Jolivet & Corriveau, 2011; Day et al., 2013; Boucher-Brossard et al., 2017). Freeze-thaw cycles can substantially decrease erosional resistance of a shore (Lawler, 1993; Gatto, 1995; Wynn, Henderson & Vaughan, 2008; Pizzuto, 2009). Frost weathering is an important process promoting shore recession (Lawler, Thorne & Hooke, 1997), and under specific conditions, itself affecting the magnitude of cliff retreat (Kaczmarek et al., 2019; Roland et al., 2021).

The Jeziorsko reservoir (central Poland) has been our testing ground for geomorphological studies, including freeze-thaw processes as a factor influencing the development of the coastal zone of an artificial body of water. The study is possible owing to high annual water level fluctuations in the reservoir averaging 5.5 m with the minimum observed during winter months. In the autumn and winter period, when the level of water fails to reach the cliff toe, the cliff is not subject to wave erosion. Hence, it is possible to separate its influence on the cliff retreat. Thanks to *in situ* field work performed in the area, such as measurements of the ground surface temperature on the cliff (Kaczmarek et al., 2019, 2021), we know that there is a relationship between the surface soil temperature on the tested cliff and the air temperature taken at the weather station located 41 km away in the city of Kalisz. In turn, measurements using Terrestrial Laser Scanning (TLS) have shown that freeze-thaw processes can be an important factor affecting the extent of cliff recession in the temperate climate zone (Kaczmarek et al., 2019).

Numerous research and reports from the Intergovernmental Panel on Climate Change (IPCC) confirm progressive climate warming. Each decade following 1980 has seen higher temperatures of the surface of the Earth than the previous one, and higher than any decade after 1850 (IPCC, 2013). Climate change observed in recent decades depending on latitude has caused an increase in the number of freeze-thaw cycles in high latitudes (Persson et al., 2007; Dyrrdal et al., 2019; Rasmus et al., 2020) and a decrease in moderate latitudes (Grossi, Brimblecombe & Harris, 2007; Kreyling & Henry, 2011). As a result, the role of freeze-thaw processes in cliff transformation, in particular the rate of its recession, has been either intensified or extinguished.

Air temperature changes and their impact on the shift of the beginning and end of the growing season in Central Europe have been given consideration in Chmielewski & Rötzer (2002), Scheifinger et al. (2003), Bielec-Bąkowska & Piotrowicz (2011), or Graczyk & Kundzewicz (2016). In their analysis of frost-free season in 1951–2010, Wypych et al. (2017) point that climate warming in Central Europe is manifested by increased minimum temperature and a declining number of freezing days. Statistically, the period without frost is also becoming longer. It must be said, though, that in this respect, due to its geographical location, Central Europe is far from homogeneous.

Therefore, we posed a question whether and what change in the role of frost processes related to freeze-thaw cycles one can expect in the vicinity of Jeziorsko reservoir in the future, depending on the projected air temperature change scenarios. We are particularly inquisitive about the impact of such changes on the pace of the cliff recession. The analysis is founded on the assessment of the current state and the direction and the rate of change in air temperature, as well as the number of freeze-thaw events in the vicinity of the reservoir. Next, the authors set to find a mathematical relationship between the mean monthly air temperature and the monthly number of days with air temperature passing 0 °C threshold.

## Materials and Methods

### Study area

**Figure 1:**
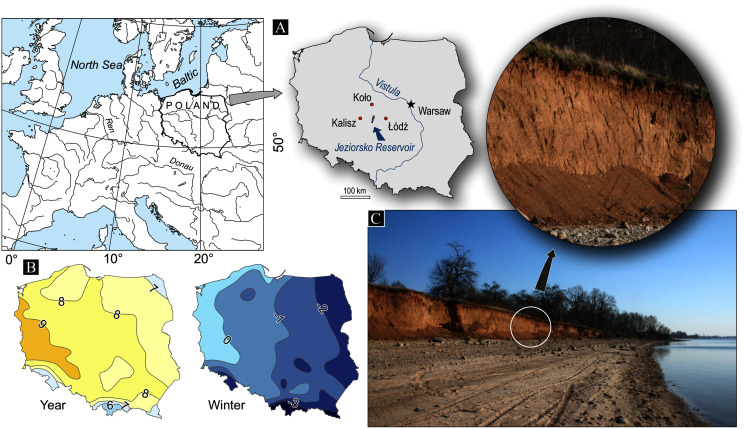
Study area (A), mean annual and mean winter temperature in Poland in the period 1981–2010 (own study based on IMWR-NRI, Climate maps of Poland, https://klimat.imgw.pl/pl/climate-maps, access 1 Feb. 2021) (B), studied cliff and effect of freeze-thaw process (C).

Created in 1991 in central Poland on the Warta river, Jeziorsko is a typical retention reservoir, ranking second in terms of area and fourth in volume in Poland (Fig. 1). Located within an old river valley on the borderline between plateaus, the reservoir is elevated to 115–118 m in the Warta valley and to 130–150 m in the plateaus (Forysiak, 2005; Kaczmarek, 2018). The slopes limiting the reservoir to the west are low and gentle, while those to the east are steeper and higher, frequently reaching heights well above 10 m. The dynamics of the eastern slopes have been subject to our detailed research (Kaczmarek, 2018; Kaczmarek et al., 2019, 2021).

The shore zone develops within Quaternary deposits, which are mainly glacial tills and sands of Riss glaciation, and to a lesser extent, sands and silty deposits of the latest glaciation (Klatkowa & Załoba, 1992a, 1992b; Załoba, 1996; Forysiak, 2005).

The Jeziorsko reservoir is located in the temperate climate zone. The mean annual air temperature for the area in 1951–2015 was +8.0 °C with the minimum of +6.0 °C and the maximum of +9.9 °C (Kaczmarek, 2018; Kaczmarek et al., 2019). The length of the period with negative mean daily air temperatures is between 70 and 80 days (Limanówka & Niedźwiedź, 1994), while the soil maximum frost depth is one meter (Polish Standard PN-81 N-03020, 1981). The study area is characterised by the country’s lowest level of precipitation with the annual totals remaining below 550 mm, nearly half of which is recorded between May and August (Kaczmarek, 2018; Kaczmarek et al., 2019). The mean annual accumulated thickness of snow cover in 1950–1989 was 640 cm, however the share of winter days without snow amounts to 50% (Niedźwiecki, 1998).

The area where the reservoir is located is among those with Poland’s lowest annual total of freeze-thaw days (FTDs) amounting to 66–71 in 1951–2018 (Kaczmarek et al., 2021). What is more, their number in the area decreased by 4 FTDs/10 years (Kaczmarek et al., 2021) over the study period.

The Jeziorsko reservoir is characterised by an annual cycle of operation with substantial water level fluctuations of up to 5.5 m. Historically, these changes ranged from 2.6 m to 5.65 m per year. Spring-time meltwater causes the level in the reservoir to increase, which is maintained over the summer months (May–September). In the autumn and early-winter months the water level gradually decreases to reach the minimum in January and February (Kaczmarek, 2018). A 15.3 km stretch of the shoreline, out of the total of 45.8 km, *i.e*. over half of the unregulated shore, is subject to coastal erosion and retreat. The cliffline recession results from the combined impact of wave erosion and subaerial processes (Kaczmarek, 2018; Kaczmarek et al., 2019).

Our TLS monitoring-based study conducted in 2014–2015 on the west-facing, 124 m-long stretch of Jeziorsko cliff face with an average height of 3.9 m, developed in frost-vulnerable silt and clay-rich sediments (the content of silt (0.002–0.075 mm) and clay (finer than 0.002 mm) reaches 73–85%; plastic limit PL = 12%, liquid limit LL = 26–29%, and plasticity index PI = 14–16%), indicates that the magnitude of recession within the studied cliff caused by freeze-thaw processes was max. 10 cm with the mean value of approx. 3 cm (Kaczmarek et al., 2019).

The 2014–2015 study coincided with the first season when, due to a changed reservoir operating regime, the level of water did not reach the cliff base. It enabled the authors to perform the assessment of the transformations within the cliff and the amount of the material accumulated at its toe during the summer period. The volume of the material deposited at the cliff toe in the winter of 2014–2015 and in the other seasons of the year suggests that freeze-thaw processes were responsible for 70% of the annual cliff recession in the absence of wave action. At the same time, this value makes up 20% of the annual cliff recession recorded in previous years when wave erosion took place (Kaczmarek et al., 2019). The studied shore stretch is not subject to transformation caused by slides or solifluction, nor are there any groundwater seeps. In the winter season, snow cover does not appear on the surface of the vertical or nearly vertical cliff face. In terms of precipitation, the study period can be classified as typical (Kaczmarek, 2018).

### Meteorological data and terminology

Depending on the scope of analysis, its purpose, timespan and data availability, various kinds of data are adopted to characterise the thermal conditions of freeze-thaw processes (Gatto, 1995; Kreyling & Henry, 2011; Nilsen et al., 2021; Kaczmarek et al., 2021).

Our analysis was performed based on the 1951–2018 mean, minimum and maximum daily air temperatures 2 meters above the ground level obtained from three weather stations closest to the studied reservoir (41 to 46 km away), and belonging to the measurement network of the Institute of Meteorology and Water Management-National Research Institute (IMWM-NRI) in Warsaw (Poland) (Fig. 1, Table 1). The selection of the weather stations was dictated by their location, the quality of the existing data and the comparability of time series.

**Table 1:** Weather stations used in the paper.

Station	Latitude	Longitude	Elevation [m a.s.l.]
Kalisz	51°46′52″	18°04′51″	140
Koło	52°11′59″	18°39′37″	116
Łódź	51°43′24″	19°23′59″	187

Based on available daily air temperatures, days with zero-crossings were identified. A day with a freeze-thaw event, referred to as a freeze-thaw day (FTD), is defined as the one on which the maximum daily air temperature is >0 °C and the minimum one is ≤0 °C. An FTD indicates the occurrence of at least one event of zero-crossing during a given day. The term “days with zero-crossings”, abbreviated DZC (Kjellström et al., 2016; Nilsen et al., 2021), is also frequently used to denote the same phenomenon. To ensure full coverage of the period in which such fluctuations appeared (autumn, winter, spring) the analysis encompassed one year from 1 August to 31 July.

### Methodology

In order to determine the characteristics of frost action conditions at the cliff surface and to test the relationship between the air temperature at 2 meters above the ground level obtained from the IMWM-NRI weather stations and the temperature at the cliff face, the authors performed their own temperature measurements in the winter season of 2014–2015. The task was carried out with a HOBO temperature logger placed on the active, plant and snow cover-free, nearly vertical cliff surface, 0.3 m below a dense herbaceous plant cover. The measurements, taken with the frequency of 10 min and accuracy of ±0.2 °C took place from 11 Dec. 2014 to 17 Mar. 2015, which coincides with two cliff face morphometry measurement series performed with a Terrestrial Laser Scanner (TLS).

The study of the direction of changes in air temperature and FTD (estimation and significance) was carried out with the non-parametric Mann-Kendall (MK) test (Hirsch, Slack & Smith, 1982) commonly used to detect a monotonic trend in environmental, climate or hydrological data series. The null hypothesis of the test assumes that when no trend is present, the measurements (data) over time are independent (*i.e*. are not serially correlated over time) and identically distributed. The alternative test hypothesis assumes that the data show a monotonic trend. The significance of the MK test was assessed at the significance level of 0.05. The analysis also used the linear trend function to make a measurable assessment of the decrease or increase in the studied indicators per unit of time (*e.g*. 1 year, 10 years). A t-test was used to assess the significance of the regression coefficient in the linear trend function. The significance of the t-test was assessed at the significance level of 0.05.

Polynomial regression was used to describe the non-linear relationship between the studied features. The least-squares polynomial approximation can easily be considered as an example of multiple linear regression.

Due to a possibly high degree of relationship between polynomial regression coefficients, it was reduced with the orthogonal polynomial method. For ease of interpretation, the results are presented in the form of a polynomial equation in its usual form. The application of polynomials results both from their properties (any continuous function on a half-open interval can be approximated with any accuracy using polynomials) and the lack of assumption on the specific functional form of the relationship being studied. The relationship between mean monthly air temperature and the monthly number of freeze-thaw events has been described with polynomial functions as had been successfully done by Ho & Gough (2006) to study similar relationships in Toronto, Canada. In our research, we applied functions of degree two to six. To assess the fit of the polynomial regression, we used the adjusted R-squared and the information criteria: Akaike Information Criterion (AIC) (Akaike, 1974) and Bayesian Information Criterion (BIC) (Schwarz, 1978). Moreover, the statistical significance of the parameters of polynomial models was checked. The fitting of data and the significance of regression were checked with the t-test. For all the tests, *p*-values smaller than or equal to 0.05 were accepted as statistically significant.

The estimated polynomial model, based on data sets from 1951/52–2017/18, provided the foundation for sketching scenarios about the future number of FTDs at a specific change in mean air temperature.

## Results

### Air temperature in the Stevenson screen *vs*. ground temperature on the cliff surface

**Figure 2:**
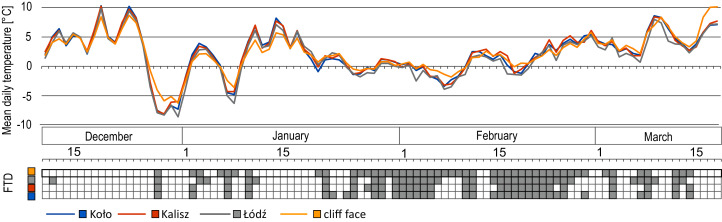
Mean daily ground temperature on the cliff face at Jeziorsko reservoir and the mean daily air temperature in Koło, Kalisz and Łódź in the winter–spring season of 2014/2015, with the number of FTDs.

The comparison of mean daily air temperatures at the three selected IMWM synoptic stations closest to the studied reservoir with the cliff surface temperature data for the period of the research in the 2014–2015 season shows a high level of similarity both in the trend and the recorded values (Fig. 2). On the other hand, the comparison of the number of FTDs determined on the basis of the maximum and minimum daily temperature on the cliff face and air temperature measurements at a height of 2 meters above ground level shows that out of 48 FTDs identified on the cliff, 30 to 39 FTDs were also recorded in Stevenson screens, *i.e*. 64–81% of those on the cliff (Fig. 2).

The notably higher number of FTDs recorded on the cliff face means that the surface layer of the ground experiences no fewer FTDs than is indicated by the air temperature measurements performed at the other stations concerned. The Kalisz-based readings display the highest level of similarity in trends and values to the results of the cliff surface temperature measurements. Additionally, each FTD identified on the basis of air temperature measurements in Kalisz was confirmed by the cliff face temperature check (Fig. 2).

### Air temperature

**Figure 3:**
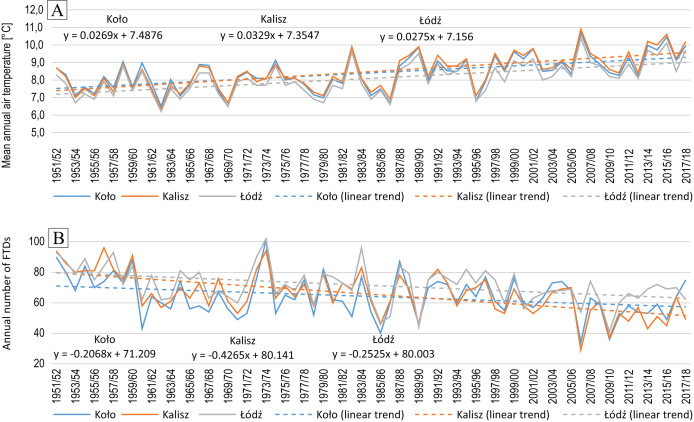
Mean annual (August–July) air temperature (A) and annual (August–July) number of FTDs (B) in Koło, Kalisz and Łódź in 1951/52–2017/18.

Mean annual air temperature for the entire study period ranged from +8.1 °C to +8.5 °C (Table 2, Fig. 3A). The results obtained from the MK test and t-test indicate a statistically significant growing trend (Fig. 3A) with the rate of change amounting to +0.27 °C/10 years in Koło, +0.28 °C/10 years in Łódź and +0.33 °C/10 years in Kalisz. During the operation of the reservoir *i.e*. 1991/92–2017/18, the mean air temperature at all these stations, was higher by approx. 1 °C as compared to the preceding period (1951–1990) (Table 2).

**Table 2:** Air temperature and number of FTDs in Kalisz, Koło and Łódź in 1951/52–2017/18.

Station	Mean annual temperature (Aug.–Jul.) [°C]	Warmest year [°C]	Coldest year [°C]	Mean annual FTDs	Annual maximum FTDs	Annual minimum FTDs
1951/1952–2017/2018						
Kalisz	8.5	10.9 (2006/07)	6.3 (1962/63)	65.6	96 (1956/57)	29 (2006/07)
Koło	8.4	10.7 (2006/07)	6.5 (1962/63) (1969/70)	64.2	101 (1973/74)	34 (2006/07)
Łódź	8.1	10.4 (2006/07)	6.2 (1962/63)	71.4	102 (1973/74)	41 (2009/10)
1951/1952–1990/1991						
Kalisz	8.0	9.9 (1982/83) (1989/90)	6.3 (1962/63)	70.7	96 (1956/57)	46 (1985/86) (1989/90)
Koło	8.0	9.9 (1989/90)	6.5 (1962/63) (1969/70)	66.0	101 (1973/74)	40 (1985/86)
Łódź	7.7	9.6 (1982/83)	6.2 (1962/63)	73.9	102 (1973/74)	44 (1989/90)
1991/1992–2017/2018						
Kalisz	9.2	10.9 (2006/07)	7.1 (1995/96)	58.2	82 (1991/92)	29 (2006/07)
Koło	9.0	10.7 (2006/07)	6.8 (1995/96)	61.5	77 (1996/97)	34 (2006/07)
Łódź	8.7	10.4 (2006/07)	6.8 (1995/96)	67.7	82 (1994/95)	41 (2009/10)

### Number of freeze-thaw days

Considering the study period, the mean annual number of days with a freeze-thaw action was similar in Koło and Kalisz *i.e*. 64.2 and 65.6, respectively. In Łódź, this figure was slightly higher and averaged 71.4 days (Table 2).

The annual totals of FTDs varied throughout the study period with a higher number recorded in the early years (Fig. 3B). The first nine of them, (1951/52-1959/60) saw a significant positive deviation from the average in the incidence of FTDs. On the other hand, the closing years (2006/07–2017/18), in the same respect, were marked by a negative deviation. The mean from the first period was 85 FTDs in Kalisz, 84 FTDs in Łódź and 79 FTDs in Koło. At the other end, in 2006/07–2017/18, the number of FTDs averaged 49 (Kalisz), 55 (Koło) and 63 (Łódź) which makes 57.6%, 69.6% and 75.0% of the initial period values respectively.

This variation is also made evident by comparing the periods before and after Jeziorsko reservoir was created. Initially, *i.e*. 1951–1990, the number of FTDs averaged 66–74, while in 1991–2018 it amounted to 58–68 (Table 2).

The change in the annual number of FTDs over the full period was significant at all the stations (Table 3), yet it was most pronounced in Kalisz (−4.2 days/10 years). In Łódź and Koło it was similar and amounted to −2.5 days/10 years and −2.1 days/10 years, respectively.

**Table 3:** Trend characteristics time series for FTDs in Kalisz, Koło and Łódź in 1951/52–2017/18.

Station	Oct.	Nov.	Dec.	Jan.	Feb.	Mar.	Apr.	Oct. - Apr.
Kalisz								
*p-value* MK test	0.0708	0.04*	0.0203*	0.0071*	0.2202	0.0041*	0.0001*	<0.0001*
Tendency [days/10ys]	−0.40	−0.50	−0.54	−0.81	−0.13	−0.93	−0.81	−4.12
*p-value* t-test	0.05	0.0645	0.0342*	0.0045*	0.6165	0.0032*	0.0001*	<0.0001*
Koło								
*p-value* MK test	0.9521	0.2652	0.5069	0.1456	0.4596	0.0582	0.0069*	0.0243*
Tendency [days/10ys]	−0.18	−0.19	−0.15	−0.39	+0.22	−0.67	−0.54	−1.91
*p-value* t-test	0.3983	0.4631	0.5392	0.1658	0.4223	0.0337*	0.007*	0.0173*
Łódź								
*p-value* MK test	0.9999	0.0324*	0.0166*	0.0337*	0.5574	0.2498	0.0063*	0.0011*
Tendency [days/10ys]	−0.06	−0.47	−0.55	−0.65	+0.26	−0.32	−0.53	−2.33
*p-value* t-test	0.7653	0.0959	0.0291*	0.0335*	0.3638	0.237	0.02*	0.0011*

**Note:**

* Statistically significant values at 0.05.

**Figure 4:**
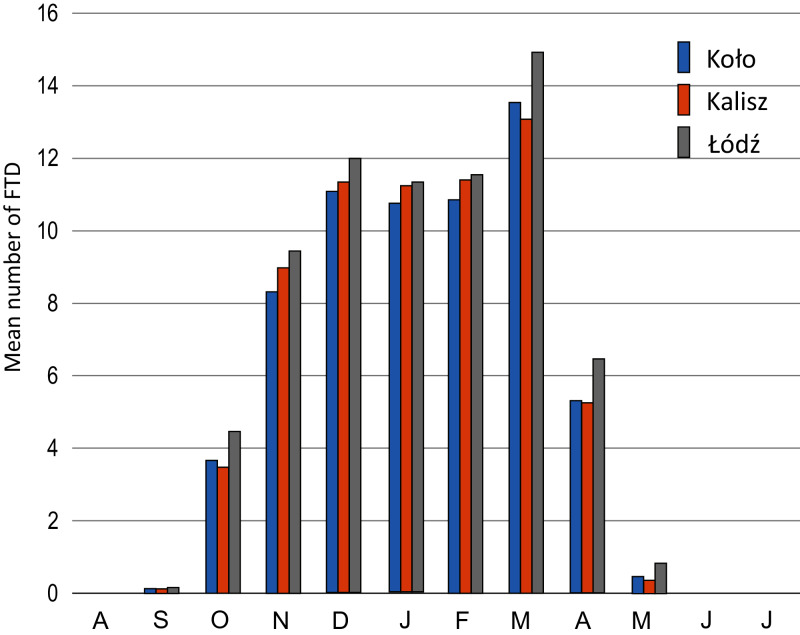
Mean number of days with a freeze-thaw event in Koło, Kalisz and Łódź in the annual cycle in 1951/52–2017/18.

The distribution of the mean number of FTDs in the annual cycle (August–July) is similar for all the stations involved. One peak is visible in March (Fig. 4), which is the month accounting for 20–21% of the annual number of FTDs in 1951–2018.

In Kalisz and Łódź the number of FTDs in individual months considerably decreased in November, December and January. All the stations also indicated a significant drop in April when the rate of change equalled −0.5 days/10 years in Łódź and Koło and −0.8 days/10 years in Kalisz (Table 3).

### Beginning, end and duration of time with FTDs

In 1951/52–2017/18, all the stations recorded the earliest FTDs in the second decade of September, and the latest in November. The days with the 0 °C transition ended the earliest in April (Łódź) and May (Koło and Kalisz), and the latest in May (Koło and Kalisz) or early June (Łódź) (Table 4).

**Table 4:** The earliest and the latest dates of the beginning and end of FTDs in Kalisz, Koło and Łódź in 1951/52–2017/18.

	Kalisz		Koło		Łódź	
	Beginning	End	Beginning	End	Beginning	End
Earliest	14.09.1973 (45)	20.03.1999 (232)	19.09.1966 (50)	21.03.1999 (233)	17.09.1959 (48)	07.04.2018 (250)
Latest	23.11.1996 (115)	20.05.1952 (293)	19.11.2000 (111)	31.05.1955 (304)	11.11.2000 (103)	01.06.1966 (305)

**Note:**

(number)–subsequent day counting subsequent days count from August 1st.

**Figure 5:**
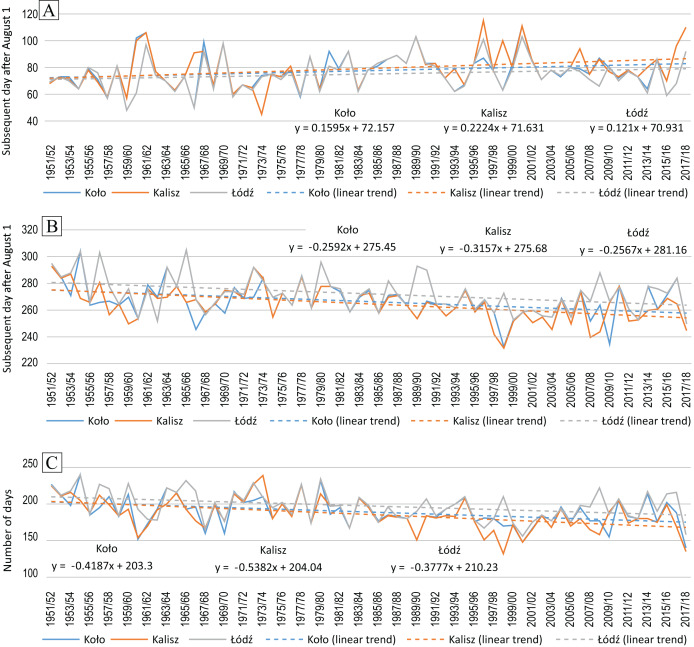
Dates of FTDs (counted as a subsequent day after August 1st): the beginning (A), the end (B), the length of the season (C) in Kalisz, Koło and Łódź in 1951/52–2017/18.

The beginning and end dates of the FTDs changed significantly (Fig. 5, Table 5). Table 5 summarises the MK test and t-test *p*-value of the series of start and end dates of the occurrence of FTDs.

**Table 5:** Trend characteristics time series for data series: beginning, end and duration of the period with FTDs in Kalisz, Koło and Łódź in 1951/52–2017/18.

	Kalisz			Koło			Łódź		
	Beginning	End	Duration	Beginning	End	Duration	Beginning	End	Duration
*p-value* MK test	0.0109*	0.0001*	<0.0001*	0.0474*	0.0027*	0.0013*	0.1450	0.0039*	0.0061*
Tendency [days/10ys]	+2.22	−3.16	−5.38	+1.60	−2.59	−4.19	+1.21	−2.57	−3.78
*p-value* t-test	0.0095*	<0.0001*	<0.0001*	0.055	0.0015*	0.0011*	0.1184	0.002*	0.0027*

**Note:**

* Statistically significant values at 0.05.

The beginning of the occurrence of FTDs was being pushed further at a rate of about −1.6 days/10 years in Koło. At the remaining stations, the mean rate of change is statistically insignificant (Table 5, Fig. 5A). On the other hand, the end of FTDs occurred sooner at a rate of approx. 3 days/10 years (Table 5, Fig. 5B).

The maximum duration of FTDs taking place was 240 days in Koło and Łodź and a similar 239 days in Kalisz. The corresponding minimum amounted to 132 days in Kalisz, 140 days in Koło and 156 days in Łódź (Fig. 5C). The change in the dates of the beginning and end of FTDs resulted in a natural consequence of narrowing down the time window in which they occurred.

### Relationship between mean monthly temperature and the number of FTDs

**Figure 6:**
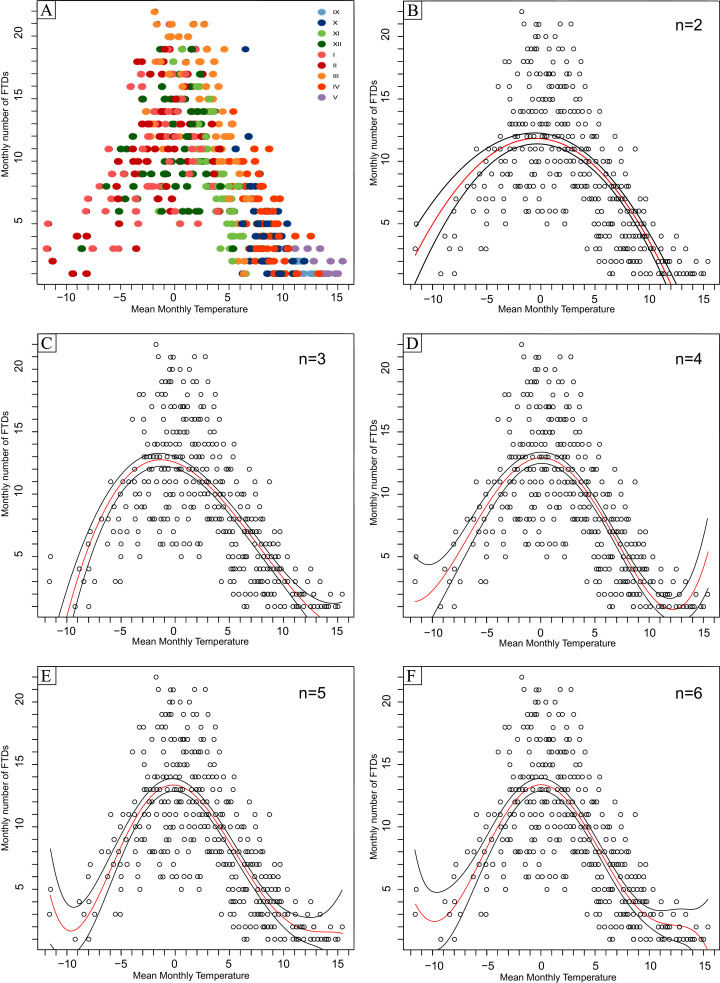
(A–F) Relationship between the mean monthly air temperature and the monthly number of days with freeze-thaw events in 1951/52–2017/18 in Koło. n, degree of polynomial model.

Figure 6 depicts the relationship between mean monthly air temperature and the monthly number of FTDs, as well as two to six-degree polynomial functions describing such a relationship as exemplified by the weather station in Koło (Fig. 6).

The spread of the number of FTDs is not symmetrical. Higher values are characteristic of the months with the mean monthly air temperature in the positive rather than negative areas. As the monthly FTDs number increases, the range of the mean monthly air temperature decreases. FTDs were recorded in the following brackets of mean monthly air temperature: −11.2 °C to +15.4 °C (Kalisz), −11.6 °C to +15.4 °C (Koło), and −11.6 °C to +17.3 °C (Łódź).

The outer “fringes” of the temperature ranges gather the months with the lowest number of FTDs, *i.e*. 1–4 in a month, which tend to be the early autumn ones, *i.e*. September, October and November, as well as the early spring months like April and May. This observation is true for all the weather stations involved in the study (*e.g*. Kalisz, Fig. 6A).

The highest monthly numbers of FTDs occur at the mean monthly air temperature oscillating around 0 °C. The highest monthly FTDs number of 24 was recorded in Łódź at the mean monthly temperature of −0.1 °C. In Kalisz and Koło, this figure counted 23 at the mean monthly temperature of +0.3 °C and 22 at −1.8 °C, respectively.

The results of polynomial fitting for the studied stations proved the 5-degree polynomial to most adequately describe the relationship between the mean air temperature and the monthly number of FTDs (Table 6). At the same time, it must be noted that the parameter of the six-degree polynomial function at the highest power was statistically insignificant at all the weather stations. Table 7 presents the polynomial equations applied to describe the relationship.

**Table 6:** Parameters of polynomial equations fitting in Kalisz, Koło and Łódź in 1951/52–2017/18.

Polynomial degree	R^2^	AIC	BIC
Kalisz			
2	0.4983	2,616.74	2,630.40
3	0.5524	2,563.37	2,584.19
4	0.5767	2,537.77	2,562.77
5	0.586	2,528.18	2,557.33
6^#^	0.5852	2,530.08	2,563.41
Koło			
2	0.4755	2,634.97	2,651.66
3	0.5249	2,588.44	2,609.30
4	0.5639	2,548.34	2,573.38
5	0.5753	2,536.65	2,565.87
6^#^	0.5757	2,537.12	2,570.51
Łódź			
2	0.4961	2,772.02	2,778.87
3	0.5497	2,716.94	2,738.00
4	0.5867	2,675.11	2,700.38
5	0.5987	2,661.35	2,690.84
6^#^	0.5998	2,661.02	2,694.72

**Note:**

# The coefficient polynomial x^6^ is statistically insignificant.

**Table 7:** Polynomial equations that most adequately describe the relationship between the mean monthly temperature and the number of FTDs in Kalisz, Koło and Łódź in 1951/52–2017/18.

Station	Polynomial
Kalisz	y = −4.729·10^−5^x^5^ + 1.043·10^−3^ x^4^ + 6.321·10^−3^ x^3^ − 2.152·10^−1^x^2^ − 1.589·10^−1^x + 1.373·10^1^
Koło	y = −4.680·10^−5^x^5^ + 1.055·10^−3^x^4^ + 6.042·10^−3^x^3^ − 2.166·10^−1^x^2^ − 8.858·10^−2^x + 1.334·10^1^
Łódź	y = −3.820·10^-5^x^5^ + 1.001·10^−3^x^4^ + 4.208·10^−3^x^3^ − 2.160·10^−1^ x^2^ + 1.316·10^−2^x + 1.417·10^1^

### Projection of the change in the number of FTDs

**Figure 7:**
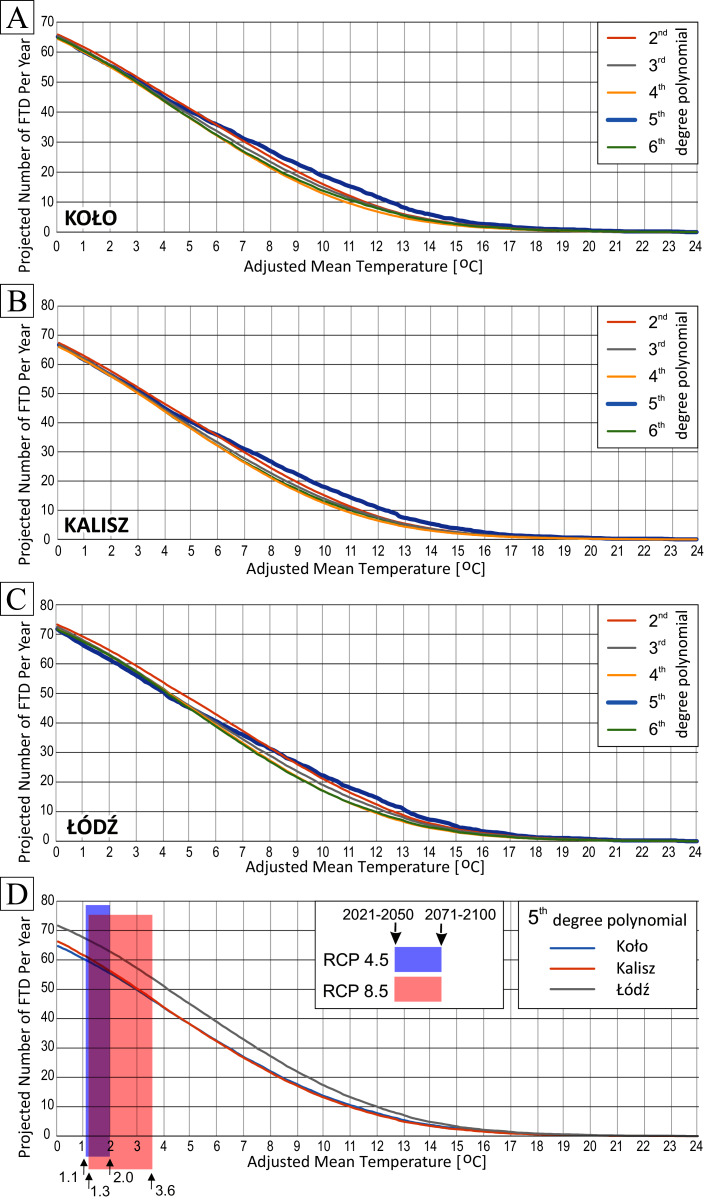
Projections of change in the number of FTDs depending on a particular temperature increase for 1951/52–2017/18 in Koło (A), Kalisz (B) and Łódź (C), projections with 5-degree polynomial (D). (D) Projections of changes in annual air temperature for the area of Poland for 2021–2051 and 2071–2100 horizons acc. to RCP4.5 and RCP8.5, based on Piniewski, Okruszko & Kundzewicz (2020).

Projections of changes in the annual number of FTDs have been presented for specific climate change scenarios according to all six polynomial models considered (Fig. 7). Calculations for each polynomial were performed in the range of mean monthly temperatures at which there were days with a freeze-thaw event. The X axis (Adjusted Mean Temperature) presents the temperature shifted by a given number of degrees and the Y axis presents corresponding projected number of FTD for given changes in temperatures. For example, the value of +1.0 °C means that in each month of the analysed period we adjusted the temperature by +1.0 °C, and for given data we calculated the corresponding projection on the number of FTDs. The value of 0 in Fig. 7 means that the mean air temperature in the model corresponds to the mean annual temperature from the 1951/52–2017/18 real data for each analysed station. Thus, the number of FTDs resulting from the model corresponds to or is close to the actual number of FTDs from the same period.

Table 8 presents mean annual numbers of FTDs recorded at the analysed stations as calculated in the period 1951/52–2017/18, as well as mean numbers of FTDs calculated with two to six-degree polynomials. Using the prepared five-degree polynomial model we calculated the annual number of days with a freeze-thaw event for the above scenario. The same procedure was repeated until the annual number of days with 0 °C transition reached the value of zero.

**Table 8:** Annual numbers of FTDs based on polynomial models and actual values for the stations in Kalisz, Koło and Łódź in 1951/52–2017/18.

Station	Actual mean for 1951/52–2017/18	Mean based on 2-degree polynomial model	Mean based on 3-degree polynomial model	Mean based on 4-degree polynomial model	Mean based on 5-degree polynomial model	Mean based on 6-degree polynomial model
Kalisz	65.64	67.37	66.76	65.98	66.24	66.24
Koło	64.17	65.85	65.35	64.38	64.70	64.73
Łódź	71.41	73.29	72.51	71.65	71.68	71.57

## Discussion

### Changes in air temperature and the number of FTDs

The information and data published in IPCC or WMO (2019) reports indicate changes in the global air temperature. The special IPCC *Global Warming of 1.5 °C* report (IPCC, 2018) informs that the mean global air temperature in the period 2006–2015 increased by 0.87 °C compared to 1850–1900 (pre-industrial period). However, the temperature growth varies geographically.

Poland (Central Europe) is in the transition zone of temperate climate, where cool continental air masses flowing in from the east clash with considerably milder maritime air masses from the west and northwest. The mean annual area air temperature in Poland in 1951–2008 was 7.9 °C (7.6 °C in the eastern part and 8.2 °C in the western one) (Limanówka et al., 2012).

Marosz et al. (2011) reported that the mean annual temperature in Poland (Central Europe) in 1951–2008 exhibited a statistically significant growing trend with the step of growth of +0.24 °C/10 years.

Mean annual (August–July) air temperature in the period 1951/52–2017/18 at all the analysed stations in the vicinity of Jeziorsko reservoir demonstrated statistically significant, positive changes of about +0.3 °C/10 years. This increase has consequently narrowed the time window for the occurrence of days with freeze-thaw activity. They began later, and the end occurred sooner, by 1.5–2.2 and 2.6–3.3 days, respectively. Also, the number of days with freeze-thaw events had been decreasing for many years by a maximum of 4.2 days/10 years in Kalisz (Fig. 3B). The shortening of the period of the occurrence of negative air temperatures, mainly as a result of earlier spring warming and later autumn cooling in the central and western parts of Poland, are also reported by Wypych et al. (2017) based on the analysis of data from 1951–2010. Graczyk & Szwed (2020) arrived at very similar conclusions following their analysis of the recent spring frost trends. By examining the series of minimum air temperatures at a height of 2.0 m and at the ground surface (0.05 m) in the period 1961–2020, *e.g*. for the Kalisz station, from which we also used the data in our research, they found that regardless of the height, a clear and statistically significant trend indicating the earlier occurrence of the last spring frost existed, with the average rate of the process ranging from 1.6 to about 3.5 days per decade.

The number of days with freeze-thaw events in the annual cycle varies significantly depending on the latitude. Wexler (1982, 1984) explained how the number of FTDs changes in particular months of the annual cycle as the latitude increases. At stations located in polar areas (extremely cold), FTDs were recorded exclusively in the summer season while those in medium latitudes with a continental climate indicated a bimodal annual distribution of FTDs with two peaks in October/November and in April. Stations at medium latitudes in the transitional climate zone recorded a high number of FTDs from November to March, with the maximum in February. At low-latitude stations (deserts and highlands), there was one peak in January. The distribution of the mean monthly number of FTDs in the annual cycle at the stations included in our study complies with the regularity observed by Wexler (1982). At the studied stations in central Poland, the largest number of FTDs occurred in the months from November to March with one culmination in March.

The relationship between mean monthly air temperature and the number of freeze-thaw events was also described by Wexler (1982, 1984) with the use of a two-parameter sine function. Schmidlin, Dethier & Eggleston (1987) and later Baker & Ruschy (1995) described the same relationship with a two-degree polynomial. Subsequently, Ho & Gough (2006) described it using two and six-degree polynomial functions. Our analyses involved the application of polynomials of the two to six-degree looking for the best fit. The obtained results of the information criteria AIC and BIC indicate that for the area of our research the analysed relationship is best represented by the five-degree polynomial function (Table 6).

Both the number and the decline in FTDs around Jeziorsko reservoir within the studied period were uneven. When the reservoir was in operation the rate of decrease in freeze-thaw processes accelerated nearly twice the rate compared to the whole study period, *i.e*. 195–2018 (Fig. 2B).

### Freeze-thaw processes as a factor intensifying cliff retreat

Frost weathering related to freeze-thaw cycles resulting from temperature fluctuations below and above 0 °C is one of the subaerial geomorphological processes influencing the rate of cliff retreat on the areas experiencing sub-zero temperatures. It causes frost-vulnerable silt and clay sediments on vertical or nearly vertical cliff faces to exfoliate and fall off exposing another layer of sediments to frost processes. Consequently, the frost front progresses into the sediments and the rate of the cliff retreat increases (Bernatchez, Jolivet & Corriveau, 2011; Day et al., 2013; Boucher-Brossard et al., 2017). This process can amount to up to 20–90% of the total annual lake or river cliff retreat being the joint effect of wave erosion and other subaerial geomorphological processes *e.g*. weathering by drying or linear erosion (Reid, 1985; Reid, Sandberg & Millsop, 1988; Gatto, 1995; Egorov & Gleizer, 2012). For Jeziorsko reservoir, this value was estimated at 20% based on the volume of sediments accumulated at the cliff toe. At the same time, the share of retreat resulting from frost processes in relation to that triggered by all the subaerial geomorphological processes at the studied cliff amounted to 70% (Kaczmarek et al., 2019). The vertical face, western exposition and the lack of the protective or insulating vegetation or snow cover on the studied cliff on Jeziorsko reservoir, along with the sediment lithology, greatly promote the efficiency of freeze-thaw processes (Isard & Schaetzl, 1998; Zhang, 2005; Wojkowski & Skowera, 2017). The influence of solar radiation on the ground, western exposure of the studied cliff, which on the northern hemisphere means more freeze-thaw cycles than is the case with eastern and northern exposures (Hall, 2004), as well as a large angle of the cliff face resulting in a greater angle of incidence of solar radiation bring about a higher number of FTDs on Jeziorsko reservoir cliff than that identified on the basis of air temperature measurements (Fig. 3). In such conditions at similar levels of moisture of the sediments subjected to freeze-thaw processes, the efficiency of each cycle is comparable, which means that greater numbers of freeze–thaw cycles may lead to greater erosion rates as they reduce cliff strength (Wynn & Mostaghimi, 2006; Thomas, Iverson & Burkart, 2009; Van Klaveren & McCool, 2010). Research has shown that in particular conditions, a single freeze-thaw cycle can reduce the soil shear strength by up to 50% (Formanek, McCool & Papendick, 1984; Van Klaveren, 1987; after Gatto, 1995). At the above assumptions, each subsequent FTD is directly translated into increased cliff face retreat, and *vice versa* a lower number of FTDs means slowing down of this process. It must be stressed, however, that the area where the reservoir is located experienced some of the lowest annual totals of FTDs in Poland in 1951–2018 (Kaczmarek et al., 2021). Moreover, during that period, their number decreased by a further 2–4 FTDs/10 years (Fig. 3B).

### Projected changes in the number of FTDs in the vicinity of Jeziorsko reservoir

Piniewski, Okruszko & Kundzewicz (2020) presented projections of changes in seasonal and annual air temperature for the area of Poland for the years 2021–2050 and 2071–2100 in relation to the reference period 1971–2000. A high (RCP8.5) and an intermediate (RCP4.5) scenario were used (Van Vuuren et al., 2011). Assuming the intermediate one (RCP4.5), the mean annual temperature in Poland will increase by +1.1 °C in the 2021–2050 horizon and +2.0 °C in 2071–2100. Assuming the high scenario (RCP8.5), the mean annual temperature in Poland will go up by +1.3 °C in 2021–2050 and by an annual average of +3.6 °C in 2071–2100.

These anticipated changes in air temperature in central Poland will be systematically reducing the number of FTDs, leading to a decline in the role of geomorphological processes related to freezing and thawing of the surface soil layer in modelling slopes with high inclination, in particular, vertical or almost vertical walls. Consequently, other subaerial processes *e.g*. weathering by drying or linear erosion, despite their similar efficiency, will start to play a greater role in the cliff transformation. At the same time, the rate of cliff retreat caused by all the subaerial processes will diminish. Still, though, this will be a slow process. With a mean yearly temperature increase of +1.1 °C, which for the RCP4.5 scenario for the area of Poland is predicted for 2021–2050 (Piniewski, Okruszko & Kundzewicz, 2020), the number of mean annual FTDs in the vicinity of Jeziorsko reservoir will drop by 6–7% in relation to 1951–2018. When the mean yearly temperature increases by +2.0 °C, as predicted for 2071–2100, the decline will reach 13–15% (Fig. 7). The RCP8.5 scenario suggests a slightly greater decline in mean annual FTDs. At the temperature increase of +1.3 °C i +3.6 °C projected for 2021–2050 and 2071–2100 (Piniewski, Okruszko & Kundzewicz, 2020) the number of FTDs will decrease by 8–9% and 25–29% respectively. Only with an increase in the mean yearly air temperature by more than +15 °C compared to the currently recorded values, could a complete disappearance of FTDs in this area be expected (Fig. 7).

In terms of the number of recorded FTDs, the whole studied period (1951–2018) could be divided into two subperiods: 1951/52–1990/91 and 1991/92–2017/18 *i.e*. when the reservoir was in operation. It is the former one which is characterised by a markedly higher number of FTDs (Table 2).

## Conclusions

In central Poland, progressing global warming results in a decrease in the number of FTDs, which indicates a gradual decline in the role of freeze-thaw activity in cliff recession. In the period 1951/52–2017/18, the number of FTDs, averaging 64–71 FTDs/year, was decreasing by 2–4 FTDs/10 years. What is more, the period over which these phenomena occur during a year was shortened. Determining the relationship between the monthly number of FTDs and the mean monthly air temperature using a polynomial function justifies the conclusion that with the progressive increase in air temperature the reduction in the number of FTDs will continue. In the next thirty years, along with the temperature increase assumed in the projections in the range of 1.1–1.3 °C (Mezghani et al., 2017; Piniewski, Okruszko & Kundzewicz, 2020) in central Poland, the number of FTDs will drop by 4.5–5.3 (6–7%) and 5.4–5.5 (8–9%), respectively. Consequently, it should also be expected that the impact of freeze-thaw processes on cliff erosion in the area will decline.

## Supplemental Information

10.7717/peerj.12153/supp-1 Supplemental Information 1 Raw data.

## Additional Information and Declarations

### Data Availability

The following information was supplied regarding data availability: The raw data are available in the “k_d_format.txt” folder from the Institute of Meteorology and Water Management - National Research Institute, Poland: https://danepubliczne.imgw.pl/data/dane_pomiarowo_obserwacyjne/dane_meteorologiczne/dobowe/klimat/. The temperature measurement stations have the following numbers: Kalisz - 351180435, Koło- 352180345 and Łódz (Łódź Lublinek) - 351190465.

## References

[ref-1] Akaike H (1974). A new look at the statistical model identification. IEEE Transactions on Automatic Control.

[ref-2] Baker DG, Ruschy DL (1995). Calculated and measured air and soil freeze-thaw frequencies. Journal of Applied Meteorology.

[ref-3] Bernatchez P, Jolivet Y, Corriveau M (2011). Development of an automated method for continuous detection and quantification of cliff erosion events. Earth Surface Processes and Landforms.

[ref-4] Bielec-Bąkowska Z, Piotrowicz K (2011). Variability of frost-free season in Poland in the period 1951–2006. Prace i Studia Geograficzne.

[ref-5] Boucher-Brossard G, Bernatchez P, Corriveau M, Jolivet Y (2017). Calculating lateral frost front penetration in a rapidly retreating cliff of fine sediments. Permafrost and Periglacial Processes.

[ref-6] Brown EA, Wu ChH, Mickelson DM, Edil TB (2005). Factors controlling rates of bluff recession at two sites on Lake Michigan. Journal of Great Lakes Research.

[ref-7] Carter CH, Guy DE (1988). Coastal erosion: processes, timing and magnitudes at the bluff toe. Marine Geology.

[ref-8] Chmielewski FM, Rötzer T (2002). Annual and spatial variability of the beginning of growing season in Europe in relation to air temperature changes. Climate Research.

[ref-9] Davidson-Arnott R (2016). Erosion of cohesive bluff shorelines. A discussion paper on processes controlling erosion and recession of cohesive shorelines with particular reference to the Ausable Bayfield Conservation Authority (ABCA) Shoreline North of Grand Bend. https://bsra.ca/download/Discussion-Paper-on-Erosion-of-Cohesive-Bluff-Shorelines-FINAL.pdf.

[ref-10] Day SS, Gran KB, Belmont P, Wawrzyniec T (2013). Measuring bluff erosion part 2: pairing aerial photographs and terrestrial laser scanning to create a watershed scale sediment budget. Earth Surface Processes and Landforms.

[ref-11] Dewez TJB, Regard V, Duperret A, Lasseur E (2015). Shore platform lowering due to frost shattering during the 2009 winter at Mesnil Val, English channel coast, NW France. Earth Surface Processes and Landforms.

[ref-12] Dyrrdal AV, Isaksen K, Jacobsen JKS, Nilsen IB (2019). Present and future changes in winter climate indices relevant for access disruptions in Troms, northern Norway. Natural Hazards and Earth System Sciences.

[ref-13] Edil TB (2010). Erosion, slope stability, prediction of future recession in actively eroding slopes. Geotechnical Engineering Journal of the SEAGS & AGSSEA.

[ref-74] Egorov IE, Gleizer IV (2012). Coastal process of the right bank of votkinsk reservoir. Vestnik Udmurskogo Universiteta, Biologia, Nauki o Ziemle.

[ref-76] Formanek GE, McCool DK, Papendick RI (1984). Freeze–thaw and consolidation effects on strength of a wet silt loam. Transactions of the ASAE.

[ref-14] Forysiak J (2005). The development of the Warta River Valley between Burzenin and Dobrów in the Late Qaternary Period. Acta Geographica Lodziensia, Łódzkie Towarzystwo Naukowe.

[ref-15] Gatto LW (1995). Soil freeze-thaw effects on bank erodibility and stability.

[ref-16] Graczyk D, Kundzewicz ZW (2016). Changes of temperature-related agroclimatic indices in Poland. Theoretical and Applied Climatology.

[ref-17] Graczyk D, Szwed M (2020). Changes in the occurrence of Late Spring Frost in Poland. Agronomy.

[ref-18] Grossi CM, Brimblecombe P, Harris I (2007). Predicting long term freeze-thaw risks on Europe built heritage and archaeological sites in a changing climate. Science of the Total Environment.

[ref-19] Hall K (2004). Evidence for freeze-thaw events and their implications for Rock Weathering in Northern Canada. Earth Surface Processes and Landforms.

[ref-20] Henry HAL (2008). Climate change and soil freezing dynamics: historical trends and projected changes. Climatic Change.

[ref-21] Hirsch RM, Slack JR, Smith RA (1982). Techniques of trend analysis for monthly water quality data. Water Resources Research.

[ref-22] Ho E, Gough WA (2006). Freeze thaw cycles in Toronto, Canada in a changing climate. Theoretical and Applied Climatology.

[ref-23] Stocker TF, Qin D, Plattner G-K, Tignor M, Allen SK, Boschung J, Nauels A, Xia Y, Bex V, Midgley PM, IPCC (2013). Climate cange 2013. The physical science basis. Contribution of Working Group I to the Fifth Assessment Report of the Intergovernmental Panel on Climate Change.

[ref-24] Masson-Delmotte V, Zhai P, Pörtner HO, Roberts D, Skea J, Shukla PR, Pirani A, Moufouma-Okia W, Péan C, Pidcock R, Connors S, Matthews JBR, Chen Y, Zhou X, Gomis MI, Lonnoy E, Maycock T, Tignor M, Waterfield T, IPCC (2018). Summary for policymakers. Global Warming of 1.5°C. An IPCC Special Report on the Impacts of Global Warming of 1.5°C above Pre-industrial levels and Related Global Greenhouse Gas Emission Pathways, in the Context of Strengthening the Global Response to the Threat of Climate Change, Sustainable Development, and Efforts to Eradicate Poverty.

[ref-25] Isard SA, Schaetzl RJ (1998). Effects of winter weather conditions on soil freezing in Southern Michigan. Physical Geography.

[ref-26] Jabro JD, Iversen WM, Evans RG, Allen BL, Stevens WB (2014). Repeated freeze-thaw cycle effects on soil compaction in a Clay Loam in Northeastern Montana. Soil Science Society of America Journal.

[ref-27] Joeckel RM, Diffendal RF (2004). Geomorphic and environmental change around a large, aging reservoir: Lake C.W. McConaughy, Western Nebraska, USA. Environmental and Engineering Geoscience.

[ref-28] Kaczmarek H (2018). Evolution of the Shore Zone of Lowland Water Reservoirs in the Conditions of Significant Water Level Fluctuations on the Example of the Jeziorsko reservoir on the River Warta. Prace Geograficzne IGiPZ PAN 256, Warsaw (in Polish). https://www.rcin.org.pl/igipz/dlibra/publication/87223/edition/67060.

[ref-29] Kaczmarek H, Tyszkowski S, Banach M (2015). Landslide development at the shores of a dam reservoir (Włocławek, Poland), based on 40 years of research. Environmental Earth Sciences.

[ref-30] Kaczmarek H, Mazaeva OA, Kozyreva EA, Babicheva VA, Tyszkowski S, Rybchenko AA, Brykała D, Bartczak A, Słowińnski M (2016). Impact of large water level fluctuations on geomorphological processes and their interactions in the shore zone of a dam reservoir. Journal of Great Lakes Research.

[ref-31] Kaczmarek H, Tyszkowski S, Bartczak A, Kramkowski M, Wasak K (2019). The role of freeze-thaw action in dam reservoir cliff degradation assessed by terrestrial laser scanning: a case study of Jeziorsko reservoir (Central Poland). Science of the Total Environment.

[ref-32] Kaczmarek H, Bartczak A, Tyszkowski S, Badocha M, Krzemiński M (2021). The impact of freeze-thaw processes on a cliff recession rate in the face of temperate zone climate change. Catena.

[ref-33] Kerguillec R (2015). Seasonal distribution and variability of atmospheric freeze/thaw cycles in Norway over the last six decades (1950–2013). Boreas.

[ref-34] Kimiaghalam N, Goharrokhi M, Clark SP, Ahmari H (2015). A comprehensive fluvial geomorphology study of riverbank erosion on the Red River in Winnipeg, Manitoba, Canada. Journal of Hydrology.

[ref-35] Kjellström E, Bärring L, Nikulin G, Nilsson C, Persson G, Strandberg G (2016). Production and use of regional climate model projections–a Swedish perspective on building climate services. Climate Services.

[ref-36] Klatkowa H, Załoba M (1992a). Detailed geological map of Poland 1 : 50 000. Sheet Warta, PIG, Warsaw (in Polish). http://bazadata.pgi.gov.pl/data/smgp/arkusze_skany/smgp0624.jpg.

[ref-37] Klatkowa H, Załoba M (1992b). Explanations to the detailed geological map of Poland 1 : 50 000. Sheet Warta, Warsaw (in Polish). http://bazadata.pgi.gov.pl/data/smgp/arkusze_txt/smgp0625.pdf.

[ref-38] Kreyling J, Henry HAL (2011). Vanishing winters in Germany: soil frost dynamics and snow cover trends, and ecological implications. Climate Research.

[ref-39] Lawler DM, Thorne CR, Hooke JM, Thorne CR, Hey RD, Newson MD (1997). Bank erosion and instability. Applied Fluvial Geomorphology for River Engineering and Management.

[ref-40] Lawler DM (1993). The measurement of river bank erosion and lateral channel change: a review. Earth Surface Processes and Landforms.

[ref-41] Limanówka D, Biernacik D, Czernecki B, Farat R, Filipiak J, Kasprowicz T, Pyrc R, Urban G, Wójcik R, Wibig J, Jakusik E (2012). Climate change and variability since the mid-20th century. Climatic and Oceanographic Conditions in Poland and the South Baltic. Expected Changes and Guidelines for the Development of Adaptation Strategies in the National Economy.

[ref-42] Limanówka D, Niedźwiedź T (1994). Mean duration of thermic winter (season with mean daily temperature below 0°C). Atlas of the Republic of Poland.

[ref-43] Marosz M, Wójcik R, Biernacik D, Jakusik E, Pilarski M, Owczarek M, Miętus M (2011). Poland’s climate variability 1951–2008. KLIMAT project’s results. Prace i Studia Geograficzne.

[ref-44] Mezghani A, Parding KM, Dobler A, Benestad RE, Haugen J, Piniewski M, Kundzewicz ZW, Hov Ø, Okruszko T (2017). Projection of temperature, rainfall and snow cover in Poland. Climate Changes and their Impact on Selected Sectors in Poland.

[ref-45] Michalowski RL, Zhu M (2006). Frost heave modelling using porosity rate function. International Journal for Numerical and Analytical Methods in Geomechanics.

[ref-46] Mostaghimi S, Young RA, Wilts AR, Kenimer AL (1988). Effects of frost action on soil aggregate stability. Transactions of the ASAE.

[ref-47] Niedźwiecki M (1998). Characteristics of the snow cover in Łódź in the years 1950–1989. Acta Universitatis Lodziensis Folia Geographica Physica.

[ref-48] Nilsen IB, Hanssen‐Bauer I, Tveito OE, Wong WK (2021). Projected changes in days with zero-crossings for Norway. International Journal of Climatology.

[ref-49] Persson G, Bärring L, Kjellström E, Strandberg G, Rummukainen M (2007). Climate indices for vulnerability assessments. Reports Meteorology and Climatology. Swedish Meteorological and Hydrological Institute, Sweden. 111. https://www.smhi.se/polopoly_fs/1.2096!/RMK111%5B1%5D.pdf.

[ref-50] Piniewski M, Okruszko T, Kundzewicz ZW (2020). Impact of the climate change on the water resources of Poland. Gospodarka Wodna.

[ref-51] Pizzuto J (2009). An empirical model of event scale cohesive bank profile evolution Earth Surface. Processes and Landforms.

[ref-52] Polish Standard PN-81 N-03020 (1981). Building grounds. Direct foundation of structures. Static Calculations and Design. Polish Committee for Standardization, Measurement and Quality (in Polish). https://docer.pl/doc/xs0118n.

[ref-53] Rasmus S, Turunen M, Luomaranta A, Kivinen S, Jylhä K, Räihä J (2020). Climate change and reindeer management in Finland: co-analysis of practitioner knowledge and meteorological data for better adaptation. Science of the Total Environment.

[ref-72] Reid JR (1985). Bank erosion processes in a cold-temperature environment, Orwell Lake, Minnesota. Geological Society of America Bulletin.

[ref-73] Reid JR, Sandberg BS, Millsop MD (1988). Bank recession processes, rates, and prediction, Lake Sakakawea, North Dakota, U.S.A. Geomorphology.

[ref-54] Roland CJ, Zoet LK, Rawling JE, Cardiff M (2021). Seasonality in cold coast bluff erosion processes. Geomorphology.

[ref-55] Scheifinger H, Menzel A, Koch E, Peter CH (2003). Trends of spring time frost events and phenological dates in Central Europe. Theoretical and Applied Climatology.

[ref-56] Schmidlin TW, Dethier BE, Eggleston KL (1987). Freeze-thaw in the Northeastern United States. Journal of Climate and Applied Meteorology.

[ref-57] Schwarz G (1978). Estimating the dimension of a model. The Annals of Statistics.

[ref-58] Spanilá T, Simeonova G (1993). Bank deformations on some water reservoirs in Bulgaria and Czechoslovakia. Acta Montana IGT AS CR, Ser. A.

[ref-59] Thomas JT, Iverson NR, Burkart MR (2009). Bank-collapse processes in a valley bottom gully, western Iowa. Earth Surface Processes and Landforms.

[ref-60] Tyszkowski S, Kaczmarek H, Słowiński M, Kozyreva E, Brykała D, Rybchenko A, Babicheva VA (2015). Geology, permafrost, and lake level changes as factors initiating landslides on Olkhon Island (Lake Baikal, Siberia). Landslides.

[ref-61] Wexler RL (1982). A general climatological guide to daily freezing conditions: frost days, ice days and freeze-thaw days. U.S. Army Corps of Engineers, Engineering Topographic Laboratories, Rep. ETL-0287, Fort Belvoir, Virginia. https://erdc-library.erdc.dren.mil/jspui/bitstream/11681/3211/1/ETL-0287.pdf.

[ref-62] Wexler RL (1984). Diurnal freeze-thaw frequencies in the high latitudes, U.S. Army Corps of Engineers, Engineering Topographic Laboratories. Rep. ETL-0364, Fort Belvoir, Virginia. https://apps.dtic.mil/sti/citations/ADA135869.

[ref-63] WMO (2019). WMO Statement on the State of the Global Climate in 2018.

[ref-64] Wojkowski J, Skowera B (2017). Relation of soil temperature with air temperature at the jurassic river valley. Ecological Engineering.

[ref-65] Wynn TM, Mostaghimi S (2006). Effects of riparian vegetation on stream bank subaerial processes in southwestern Virginia, USA. Earth Surface Processes and Landforms.

[ref-66] Wynn TM, Henderson MB, Vaughan DH (2008). Changes in streambank erodibility and critical shear stress due to subaerial processes along a headwater stream, southwestern Virginia, USA. Geomorphology.

[ref-67] Wypych A, Ustrnul Z, Sulikowska A, Chmielewski FM, Bochenek B (2017). Spatial and temporal variability of the frost-free season in Central Europe and its circulation background. International Journal of Climatology.

[ref-75] Van Klaveren RW (1987). Hydraulic Erosion Resistance of Thawing Soil, Ph.D dissertation.

[ref-68] Van Klaveren RW, McCool DK (2010). Freeze-thaw and water tension effects on soil detachment. Soil Science Society of America Journal.

[ref-69] Van Vuuren DP, Edmonds J, Kainuma M, Riahi K, Thomson A, Hibbard K, Hurtt GC, Kram T, Krey V, Lamarque J-F, Masui T, Meinshausen M, Nakicenovic N, Smith SJ, Rose SK (2011). The representative concentration pathways: an overview. Climatic Change.

[ref-70] Załoba M (1996). Traces of the Warcian glacier oscillation in the eastern part of the Warta and Prosna interphase. Acta Geographica Lodziensia.

[ref-71] Zhang T (2005). Influence of the seasonal snow cover on the ground thermal regime: an overview. Reviews of Geophysics.

