# Supplementary material for: Measured and predicted freeze-thaw days frequencies in climate change conditions in central Poland
Source: PeerJ. 2021 Oct 26;9:e12153. doi: 10.7717/peerj.12153 (PMC8555500; doi:10.7717/peerj.12153)
Supplement: Supplemental Information 1 [file peerj-09-12153-s001.docx]

Raw data are available on the server of Institute of Meteorology and Water Management, Poland. Data may be used free of charge for scientific purposes. In the paper we used the following file:" k_d_format.txt". The temperature measurement stations have the following numbers: Kalisz - 351180435, Koło- 352180345 and Łódz (Łódź Lublinek) - 351190465.

Link: https://danepubliczne.imgw.pl/data/dane_pomiarowo_obserwacyjne/dane_meteorologiczne/dobowe/klimat/
